# Metabolic Bone Disease of Prematurity: Diagnosis and Management

**DOI:** 10.3389/fped.2019.00143

**Published:** 2019-04-12

**Authors:** Maria Felicia Faienza, Elena D'Amato, Maria Pia Natale, Maria Grano, Mariangela Chiarito, Giacomina Brunetti, Gabriele D'Amato

**Affiliations:** ^1^Pediatric Section, Department of Biomedicine and Human Oncology, University of Bari A. Moro, Bari, Italy; ^2^Department of Electric and Electronic Engineering, City University of London, London, United Kingdom; ^3^Neonatal Intensive Care Unit, Di Venere Hospital, Bari, Italy; ^4^Section of Human Anatomy and Histology, Department of Emergency and Organ Transplantation, University of Bari A. Moro, Bari, Italy; ^5^Section of Human Anatomy and Histology, Department of Basic Medical Sciences, Neurosciences and Sense Organs, University of Bari A. Moro, Bari, Italy

**Keywords:** metabolic bone disease, prematurity, osteopenia, mineral supplementation, total parenteral nutrition, enteral feeding

## Abstract

Metabolic Bone Disease (MBD) of prematurity is a multifactorial disorder commonly observed in very low birth weight (VLBW, <1,500 g) newborns, with a greater incidence in those extremely low birth weight (ELBW, <1,000 g). MBD is characterized by biochemical and radiological findings related to bone demineralization. Several antenatal and postnatal risk factors have been associated to MBD of prematurity, although the main pathogenetic mechanism is represented by the reduced placental transfer of calcium and phosphate related to preterm birth. The diagnosis of MBD of prematurity requires the assessment of several biochemical markers, radiological, and ultrasonographic findings. However, the best approach is the prevention of the symptomatic disease, based on the screening of subjects exposed to the risks of developing MBD. Regarding the subjects who need to be screened, there is a substantial agreement on the potential risk factors for MBD. On the contrary, different recommendations exist on the diagnosis, management and treatment of this disorder of bone metabolism. This review was aimed at: (1) identifying the subjects at risk for MBD of prematurity; (2) indicating the biochemical findings to take in consideration for the prevention of MBD of prematurity; (3) suggesting practical recommendations on nutritional intake and supplementation in these subjects. We searched for papers which report the current recommendations for biochemical assessment of MBD of prematurity and for its prevention and treatment. The majority of the authors suggest that MBD of prematurity is a disease which tends to normalize overtime, thus it is not mandatory to mimic the rate of mineral fetal accretion through parenteral or enteral supplementation. The optimization of total parenteral nutrition (TPN) and the early achievement of a full enteral feeding are important goals for the prevention and management of MBD of prematurity.

## Introduction

Metabolic Bone Disease (MBD) of prematurity is a disorder of bone health whose distinctive features are represented by hypophosphatemia, hyperphosphatasemia and late onset of radiological findings of bone demineralization (1, 2). It is frequently observed in newborns <28 weeks of gestation, occurring in 16–40% of very low birth weight (VLBW, <1,500 g) and extremely low birth weight (ELBW, <1,000 g) infants, with a peak at 4–8 weeks of postnatal age (3). The clinical signs of MBD of prematurity appear between 5 and 11 weeks of life, and are characterized by an increased work of breathing, due to chest wall instability caused by softening or fractures of ribs, an enlargement of the cranial sutures, frontal bossing, rickets, fractures, and postnatal growth failure (4, 5).

### Fetal and Neonatal Bone Homeostasis

Bone mineralization begins during embryonic phase of human development, but the large part of this process occurs in the third trimester of gestation, when the 80% of the mineral content is stored. By the 25 week of gestation the mineral accretion is about 60 mg per day, and increase to more 300 mg per day between the 35 and 38th weeks (6).The osteoblasts produce the organic bone matrix for deposition of calcium and phosphate, with a progressively expansion of bone volume through an increase in the trabecular thickness. An impaired bone remodeling, with an increased osteoclastogenesis, has been observed in subjects affected by genetic and metabolic diseases (7, 8).

**Figure 1:**
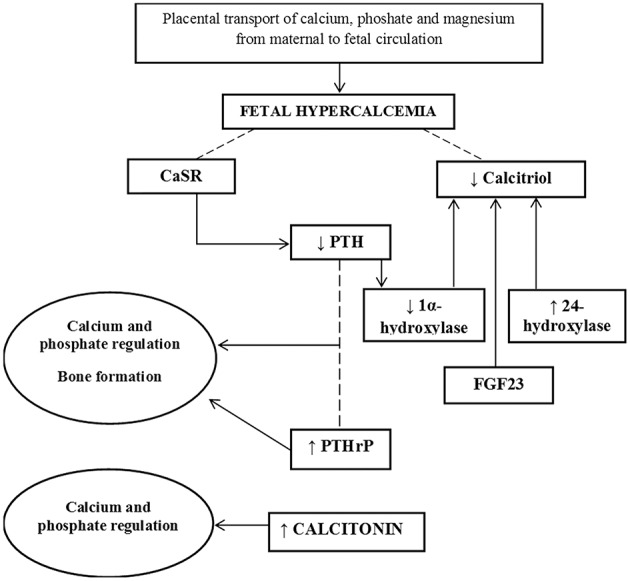
Diagram of mechanisms involved in fetal bone mineral accretion.

The function and activity of osteoblasts and osteoclasts are influenced by the availability of minerals before birth. The placenta provides calcium, phosphate and magnesium by an active transport from the maternal circulation, even in the presence of low levels of these minerals, while the kidneys and the gut have a poor role for the fetus. This fetal “hypercalcemia” is necessary for an adequate skeletal formation (6). The high fetal-maternal gradient of minerals is associated with low levels of parathyroid hormone (PTH), calcitriol, and the sex steroids, as well as high levels of calcitonin and PTH-related protein (PTHrP) (Figure 1). In particular, the calcium sensing receptors (CaSR) maintain PTH suppressed in response to the high fetal serum calcium levels. The low fetal calcitriol levels are likely due to suppression of the fetal renal 1α-hydroxylase due to low PTH levels, fibroblast growth factor-23 (FGF23), high calcium and phosphate levels, and increased activity of the catabolic enzyme 24-hydroxylase. High placental 24-hydroxylase activity causes the conversion of 25-hydroxyvitamin D to 24,25-dihydroxyvitamin D which cannot be converted to calcitriol (9). Although placental-derived human chorionic gonadotropin and the fetal pituitary gonadotropins can stimulate sex steroid production by the gonads, serum levels of testosterone and estradiol remain low until the end of the gestation in both sexes. Calcitonin is produces in the fetal thyroid and placenta, and its levels in the fetus are about twice those in the maternal circulation.

The high calcitonin levels favor the mineral deposition. PTHrP is produced by chondrocytes and perichondrial cells and it has an additive role with PTH in the regulation of the fetal calcium and phosphate. It is also produced within preosteoblasts and osteoblasts and stimulates bone formation (6).

After birth, there are rapid changes in mineral supply, hormonal environment and mechanical stress.

The placental source of minerals is interrupted, and breathing determines an increase in blood pH, with a consequent decline in serum and ionized calcium over the first 12–24 h.

These events stimulate the bone remodeling causing an increased bone resorption and a decreased bone density. Premature infants lack of the stage of greater intake of minerals during gestation and are particularly sensible to the postnatal modifications and, in addition, are exposed to risk factors of reduced bone mineralization.

### Risk Factors for MBD of Prematurity

In the Table 1 are shown antenatal and postnatal risk factors for MBD of prematurity.

**Table 1:** Antenatal and postnatal risk factors of MBD of prematurity.

**Antenal**	**Postnatal**
Placental insufficiency	Prolonged TPN > 4 weeks
Preeclampsia	Bronchopulmonary dysplasia
Chorioamnionitis	Necrotizing enterocolitis
Neuromuscolar disorders, intraventricular hemorrhage, periventricular leukomalacia	Liver disease
Genetic polymorphisms (vitamin D receptor, estrogen, collagen alpha I)	Renal disease
Male gender	Medications (loop diuretics, methylxanthines, glucocorticoids)

The most of placental transfer of calcium and phosphate occurs in the third trimester of gestation with a peak at 34 weeks (10). Pathological conditions which impair placental macro and micronutrients transfer, such as preeclampsia, intrauterine growth restriction, and chorioamnionitis are associated with an increased risk of MBD in preterm infants (3).

Data from animal models and observational studies in humans have demonstrated that calcitriol is not required to regulate serum mineral levels during the fetal life, as severe vitamin D deficiency and absence of vitamin D receptor or 1α-hydroxylase do not impair serum calcium and phosphate concentrations (9). However, clinical trials have shown that vitamin D supplementation of pregnant women reduces the risk for preeclampsia and gestational diabetes which represent risk factors for MBD (11, 12). Male gender and polymorphisms of vitamin D receptor, estrogen receptor, and collagen alpha 1 genes have also been indicated as risk factors for MBD in preterm infants (13).

After birth, the role of mineral intake of calcium, phosphate and vitamin D in the etiology of MBD of prematurity is still debated. Some studies reported that ELBW infants <30 weeks of gestation who had lower weekly intake of calcium, phosphate, vitamin D, and proteins during the first 8 weeks of life, developed MBD (14), whereas other studies have not found this correlation in the same cohort of subjects (1), and in infants <1,500 gr recruited independently from gestational age (15).

It has been demonstrated that newborns fed exclusively with breast milk showed lower levels of phosphate than those receiving special formulas or mineral supplementation (16). In addition, preterm infants fed with unfortified human milk present rickets in 40% of the cases, compared to the 16% of those fed with special formulas (17). It has also been demonstrated that phosphate supplementation improves the biochemical markers of MBD in a cohort of preterm infants with low gestational age and birth weight more than in a cohort of preterm newborns with higher gestational age and birth weight (18). A less effective intake of calcium and phosphate occurs in infants with poor tolerance to enteral nutrition and who require total parenteral nutrition (TPN) >4 weeks (19). The adverse effects related to prolonged TPN include the possibility of aluminum contamination and the risk of mineral precipitation in the solution due to the small volumes.

Common neonatal morbidities, such as sepsis, chronic lung disease (CLD) (20), acidosis, necrotizing enterocolitis, cholestatic jaundice, and long-term treatments with diuretics and glucocorticoids can impair bone remodeling by reducing osteoblast proliferation, stimulating osteoclast activity, decreasing calcium absorption, and increasing calcium renal excretion (1, 20–23). An impaired osteoblast activity due to bilirubin and bile acids has been reported in experimental studies (24). Moreover, the lack of mechanical stimuli due to fetal movements against the uterine wall, as in presence of muscular disorders and paralysis, may contribute to decrease the bone formation (25).

### Diagnosis

There are no specific diagnostic methods for MBD of prematurity. The clinical findings appear late and sometimes the diagnosis is not carried out. Indeed, it is necessary to screen the subjects who are at risk to develop MBD.

#### Serum Biochemical Markers

The assessment of serum biochemical markers is useful for early detection of mineral deficiency (third week of life). However, none of the bone metabolism markers, such as calcium, phosphate, alkaline phosphatase (ALP), PTH, and vitamin D alone can be considered specific of MBD of prematurity.

#### 1 – Calcium

The assessment of serum calcium levels is not a reliable screening tool because newborns can maintain normal calcium values despite a bone calcium loss. Furthermore, serum calcium levels may also be affected by other disorders such as phosphate depletion and hypophosphatasemia.

#### 2 – Phosphate

Hypophosphatemia is the earliest marker of disrupted mineral metabolism, occurring 7–14 days after birth. Serum phosphate levels lower than 3.6 mg/dl (1.16 mmol/L) in newborns exclusively maternal breastfed suggest the depletion of the mineral content and indicate a greater risk for MBD development (26). Serum phosphate levels <5.6 mg/dl (<1.8 mmol/L) have been strongly associated with the presence of radiological evident rickets in preterm infants with a mean gestational age of 30.3 weeks (range 24.7–33.0 weeks) and a mean birth weight of 1,490 g (range 735–2,250 g) (27).

#### 3 – ALP

ALP is a bone turnover marker which physiologically increases over the first 3 weeks of life and reaches a peak at 6–12 weeks of age (28). There are at least four ALP isoenzymes, encoded by four genes: 3 tissue non-specific alkaline phosphatase (TNSAP) (intestinal, placental, and germ cell), and the ubiquitous TNSALP especially abundant in the liver, bone and kidney, but also expressed in the brain, particularly in the cortical sensory areas. ALP levels >500 IU/L are suggestive of impaired bone homeostasis and values >700 IU/L are associated with bone demineralization, despite the absence of clinical signs (28, 29). ALP levels higher than 900 IU/L in preterm infants <33 weeks of gestational age, associated with serum phosphate levels persistently lower than 5.6 mg/dL (<1.8 mmol/L), have a diagnostic sensitivity and specificity of 70 and 100%, respectively (27). An X-ray of the wrist and/or knee has been suggested in VLBW infants when 2 values of ALP measured at least 1 week apart exceed 800 IU/L (4). Viswanathan et al. have shown that ALP level >500 IU/L in ELBW infants <30 weeks of gestation is associated with MBD (14). Of the 230 infants included in the study, 71 (30.9%) developed radiological evidence of MBD of which 24/71 (33.8%) showed spontaneous fractures. The differences of the cut-off between the above studies could depend on the selection of patients who were VLBW with higher gestational age in the studies from Backstrom and Chan, differently from those selected by Viswanathan who were an omogenous population of ELBW <30 weeks of gestation. The assessment of serum phosphate and ALP has been recommended weekly or biweekly (30, 31).

#### 4-Other Biomarkers

Serum PTH levels >100 pg/ml may suggest ELBW neonates at risk for MBD (3, 32). High PTH levels indicates not only a secondary hyperparathyroidism but, in association with the kidney tubular reabsorption of phosphate (TRP), can discriminate the underlying cause of hypophosphatemia. A low TRP with a high PTH would suggest a calcium deficiency, while a high TRP with low or normal PTH would indicate phosphate deficiency (3). Serum osteocalcin (OC), a protein of the bone matrix, is a marker of osteoblastic activity, partially regulated by 1,25-dihydroxyvitamin D levels. In presence of high bone turnover, OC levels are increased. However, despite its specificity, there is no a clear relationship between serum OC levels and bone mineral content in the first 4 months of life (33). It has been demonstrated that serum PTH levels might predict a reduction of bone mineral content in preterm infants who have reached the at term age, while urinary phosphate excretion and OC might be useful markers to predict a low bone mineralization at 3 months of corrected age (34).

#### Urinary Biomarkers

Urinary calcium and phosphate excretion have also been indicated as biomarkers of postnatal skeletal mineralization. Hypophosphatemia, the most common biochemical alteration associated with MBD of prematurity, causes reduced PTH release which increases renal tubular phosphate reabsorption. Decreased phosphate also directly stimulates renal tubular synthesis of vitamin D which increases intestinal calcium absorption. Thus, phosphate deficiency interferes with calcium balance, leading to hypercalcemia, hypercalciuria, and nephrocalcinosis (3). Infants born <28 weeks of gestation have a lower phosphate threshold value compared to other preterm newborns, resulting in elevated urinary phosphate excretion even in the presence of low phosphate levels. The normal range of TRP is 78–91% and a value above 95% is a significant marker of insufficient phosphate supplementation (3, 30). Tubular phosphorus reabsorption is calculated according to the following formula:

[1-(urinary phosphorus/urinary creatinine × serum creatinine/serum phosphorus)] × 100.

Likewise, urinary calcium or phosphate to creatinine ratios may also be useful as biomarkers for MBD, although these ratios are highly dependent on the dietary intake and are also affected by the administration of drugs such as furosemide or theophylline (30).

#### Radiological Markers

The instrumental diagnosis of bone impairment in preterm infants remains a difficult challenge.

X-rays are not reliable at early stage of bone disease due to the absence of significant demineralization or fractures (35). Bone mineralization must be reduced by 20–40% to be identifiable and usually it occurs later in life (31).

The Koo's score describes the radiological alterations (36):

-Grade 1: presence of bone rarefaction;
-Grade 2: presence of bone rarefaction associated with metaphyseal alterations, shadow, and subperiosteal bone formations;
-Grade 3: associated with the presence of spontaneous fractures.

Dual energy X-ray absorptiometry (DEXA) is the gold standard technique to assess bone mineral density (BMD) (37), adaptable to preterm infants (38). DXA expresses the bone calcium content as grams of hydroxyapatite per centimeter squared. The method implies the use of low ionizing radiation (effective dose, 0.001 mSv; <0.1 mrem), and the preferred target regions in neonates are the lumbar spine, the forearm and the calcaneus. A BMD >0.068 g/cm^2^, evaluated in a cohort of preterm infants <31 weeks (birth weight <1,500) at discharge, has been associated to a low probability of developing MBD of prematurity (38). However, the instrumental dimensions, the time employed for imaging and movement artifacts limit the widespread use of this technology in preterm and at term infants. Backstrom et al. found that the association of ALP serum levels >900 IU/L and phosphate <1.8 mmol/L indicates a low BMD with sensitivity and specificity of 100% and 70%, respectively, compared to DXA measurements, in VLBW and ELBW infants <33 weeks of gestation and mean birth weight of 1,490 g. This correlation was not found in a prospective study performed in a cohort of infant <32 weeks with a mean birth weight of 1,129 g (39). In these subjects ALP and phosphate were measured weekly from 1 week of age until 37 weeks of gestational age, and no associations were observed between either ALP or serum phosphate and bone mineralization at term.

Quantitative Ultra Sound (QUS) measures both bone mineral content and organic matrix (40). It is an inexpensive and portable modality of investigating MBD of prematurity, usually performed on the tibia. Two parameters can be evaluated by QUS: speed of sound (SOS) and bone transmission time (BTT). In some studies metacarpus BTT was used to reduce the effect of soft tissue respect to SOS (41–43). Altuncu et al. (44) observed that tibial Z scores of preterm infants (<33 weeks, mean birth weight 1,650 g) evaluated at term-corrected age were significantly lower than the Z scores at first postnatal week of life, suggesting that the decreased bone mineralization in premature infants occurs in the early postnatal period. In this study, serum ALP levels was inversely correlated to tibial Z scores in preterm infants at term-corrected age. In particular, in subjects with ALP >900 IU/L, tibial Z scores were significantly lower than infants with ALP <900 UI. Rack et al. (45) evaluated bone quality by QUS assessment in 172 preterm and at term infants with a gestational age ranging between 23 and 42 weeks (mean 33.8 ± 5.0) and a birth weight ranging from 405 to 5,130 g (mean 2,132 ± 1,091 g). The QUS parameters assessed in the first week of life correlated significantly with gestational age and birth weight. The evaluation of bone quality at 40 weeks of age showed significantly lower QUS parameters than at term infants. Furthermore, also in this study there was a significant correlation of QUS with ALP, calcium, phosphate and vitamin D.

In another study performed in preterm infants with mean gestational age of 27.54 ± 1.97 weeks and mean birth weight of 902.83 ± 216.06 g, metacarpus BTT correlates with serum phosphate but not with ALP values (35). Thus, considering the above studies, QUS assessment may have a significant role in monitoring bone health in preterm infants. However, further studies are needed to individuate which biochemical alterations could better correlate with QUS parameters.

## Prevention and Treatment

**Figure 2:**
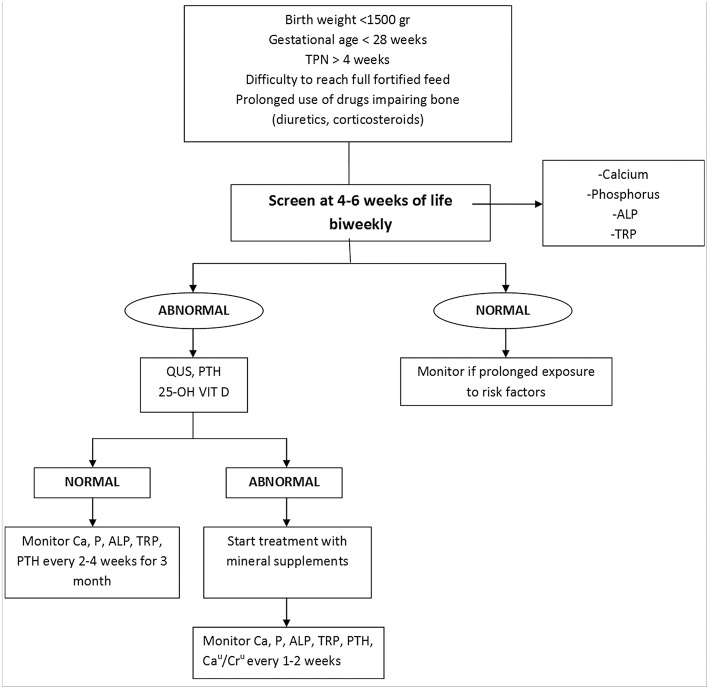
Flow chart for the diagnosis and management of MBD of prematurity.

To prevent MBD of prematurity is necessary to avoid the mentionated postnatal risk factors. The primary prevention in VLBW infants consists in improving nutrition, specifically calcium, phosphate, and vitamin D intake, and to limit the chronic use of diuretics and methylxanthines that reduce mineral stores and glucocorticoids which enhance bone resorption. Among those infants who are at high risk for MBD a biweekly monitoring of biochemical markers should be performed (**see** Figure 2).

The current recommendations for minerals and vitamin D intakes are shown in the Table 2.

**Table 2:** Calcium, phosphate and vitamin D supplementation in preterms <1,500 gr.

	**TPN first weeks**	**TPN after first weeks (fluid intake 140–150 ml/kg/day)**	**Full enteral feeding (breast milk/formula)**
Calcium	40–120 mg/kg/day (1–3 mmol/kg/day)	75–90 mg/kg/day (1.8–2.2 mmol/kg/day)	140–160 mg /100 kcal (AAP) 70–140 mg/100 kcal (ESPGHAN)
Phosphate	31–71 mg/kg/day (1.0–2.2 mmol/kg/day)	60–70 mg/kg/day (1.9–2.2 mmol/kg/day)	95–108 mg/100 kcal (AAP) 50–86 mg/100 kcal (ESPGHAN)
Vitamin D	160–280 IU/day	160–280 IU/day	200–400 IU/day (AAP)

Appropriate calcium and phosphate intake must be guaranteed with TPN, augmented during transition to enteral feeding and prolonged during the full enteral nutrition.

The solubility of calcium and phosphate in the bags of TPN is influenced by temperature, amino acid concentrations, glucose and lipids and the pH of the solution. Pereira-da-Silva et al. showed that high early calcium and phosphate intake by TPN can prevent bone strength impairment in preterm infants with a mean gestational age of 29.6 weeks and birth weight of 1,262 g, within the first weeks after birth. Current practice recommendations vary from 40 to 120 mg/kg/day (1–3 mmol/kg/day) for calcium and from 31 to 71 mg/kg/day (0.9–2.2.) for phoshate (46). When the TPN reaches the fluid intake of 150 ml/Kg/day, the calcium and phosphate supply should be of 75–90 mg/Kg/day (1.8–2.2 mmol/kg/day) and 60–70 mg/Kg/day (1.9–2.2 mmol/kg/day), respectively, with a ratio ranging from 1.5 to 1.7:1, to ensure a higher mineral accretion (47). The introduction of proteins and calories in TPN with a high intake in the first days, associated with an early introduction of enteral feedings have become commune practice in neonatal intensive care units (NICU). One effect of the higher protein intake is an increased cellular uptake of phosphate (48, 49). In preterms on full enteral feeding, the absorption of calcium ranges between 40% (for formula) and 70% (for breast milk), and that of phosphate between 60 and 95%.

The recommended oral daily intake of calcium varies between 140 and 160 mg calcium/100 kcal (American Academy of Pediatrics-AAP) (50, 51) to 70–140 mg/100 kcal (European Society of Pediatric Gastroenterology and Nutrition-ESPGAN) Similarly, recommendations for phosphate intake ranges from 95 to 108 mg/100 kcal (AAP) to 50–87 mg/100 kcal (ESPGAN) (50, 52, 53). Breast human milk provides inadequate protein and mineral content. Fortified human milk and special preterm formulas have been proposed to guarantee the needs of the growing preterm infant. The employ of human milk fortifiers and preterm formulas is recommended at least until 36 weeks' gestational age and/or 2,000 g. Phosphate supplementation should be considered for values < 4 mg/dl (1 mmol/L), but can be considered if values fall below 5.5 mg/dl (1.3 mmol/L), especially if associated with hyperphosphatasemia, to promote bone mineralization and to prevent hypercalciuria (51). There are several phosphate formulations which combine phosphate with salts, e.g., potassium acid phosphate, sodium phosphate and Joulie's phosphate. Potassium phosphate which can be used both intravenous or orally is the preferred form of phosphate supplementation because of gut intolerance due to other phosphate salt preparations. Serum calcium levels normally remain normal or high due to a compensatory secondary hyperparathyroidism. Calcium supplementation can be considered in the presence of secondary hyperparathyroidism and low TRP. Some studies have shown that a calcium/phosphate ratio of 2:1 is sufficient; this value allows avoidance nephrocalcinosis, extraskeletal calcifications, and excessive accumulation of calcium and phosphate in bone (3). The timing of vitamin D-dependent absorption of calcium and phosphate in preterm newborns is unknown. Thus, there are several concerns on the recommended intake of vitamin D in preterms. These doubts are expressed in the different recommended doses of the Nutrition Societies. A daily intake of 800–1,000 IU/day would improve serum 25(OH)D levels and the calcium absorption rate (54). Alizadeh Taheri et al. (55) demonstrated that the supplementation of 200–400 IU/day of vitamin D is sufficient in preventing osteopenia of prematurity as 1,000 IU/day. Isojima et al. (56) found no statistically significant differences in the growth of two groups of VLBW infants with MBD whether they received standard vitamin D supplementation since birth or not. Thus, further researches on vitamin D supplementation are needed. Nowadays, the AAP recommendations to provide 200–400 IU/day are accepted (51).

A systematic review of literature showed that physical activity including a protocol of passive range of motion (ROM) and joint compression performed 5–15 min daily for 4–8 weeks can improve bone mineralization in preterm infants, as demonstrated through QUS and biochemical markers of bone turnover (57). However, longitudinal studies on larger sample are needed to determine long-term outcomes of physical activity on bone health in the preterm infants.

## Post-discharge

In exclusively breastfed VLBW infants the measurement of serum ALP levels is suggested 2–4 weeks post discharge, and if ALP values are higher than 800–1,000 IU/L, mineral supplementation is needed (51). Post-discharge preterm formulas or breast milk fortification are indicated until 40–52 weeks post-conceptional age or up to 6 months in presence of low growth velocity. The duration of mineral supplementation is still debated. It is know that newborns who are fed with preterm formulas have bone mineralization comparable to that of at term infants around 6 months of age. Alternatively, preterm infants who are fed with human milk do not attain comparable bone mineralization until 2 years of life.

## Conclusion

Preterm infants on long-term diuretic or corticosteroid treatment, and those with neuromuscular disorders or brain injuries are at high risk of developing MBD of prematurity. To screen in the asymptomatic phase the neonates at risk of MBD, is useful to assess biochemical markers of bone metabolism and to evaluate bone quality by DEXA or QUS. The optimization of TPN and the reduction of the duration of TPN, the success of early achievement of full enteral feeding are important goals for the prevention and management of MBD of prematurity.

## Author Contributions

MF and GD wrote the manuscript and critally revised it. MN and MC performed medline research and selected the literature. MG critically revised the manuscript. GB focalized on risk factors for MBD of prematurity. ED critically reviewed the entire manuscript focusing on the sensitivity and specificity of the various diagnostic methods described in the review.

### Conflict of Interest Statement

The authors declare that the research was conducted in the absence of any commercial or financial relationships that could be construed as a potential conflict of interest.
